# Demographic and Functional Consequences of Secondary Host Selection in a Facultative Autoparasitoid, *Encarsia sophia* (Hymenoptera: Aphelinidae)

**DOI:** 10.3390/insects16111165

**Published:** 2025-11-14

**Authors:** Siteng Zhang, Xiaocong Wang, Jing Wang, Shuli Gao, Zhiqi Zhang, Yuning Li, Nicolas Desneux, Junjie Zhang, Yue Zhao, Changchun Ruan

**Affiliations:** 1 Jilin Provincial International Cooperation Key Laboratory for Biological Control of Agricultural Pests, Jilin Engineering Research Center of Biological Control, Institute of Biological Control, Jilin Agricultural University, Changchun 130118, China; stzhang0130@163.com (S.Z.);; 2 Université Côte d’Azur, INRAE, UMR ISA, 06000 Nice, France

**Keywords:** host feeding, life table, biological control, hyperparasitoid fitness, mass rearing, *Encarsia formosa*

## Abstract

To evaluate the impact of secondary host selection by the autoparasitoid *E. sophia* on the fitness and biological control potential of its offspring, we compared the demographic traits, parasitism capacity, and host-feeding rates of populations reared on different secondary hosts: the heterospecific *E. formosa* and the conspecific *E. sophia*. Analyses conducted with TWOSEX-MSChart, CONSUME-MSChart, and TIMING-MSChart showed that the population reared on *E. formosa* and *E. sophia* as secondary hosts. The *E. sophia* population reared on *E. formosa* exhibited significantly shorter developmental times, extended adult longevity, and enhanced female reproductive output, characterized by higher fecundity and longer oviposition periods than the conspecific-reared group. This group also displayed superior host consumption, accelerated population growth, a shorter mean generation time, and improved biocontrol efficacy. These findings underscore the importance of secondary host optimization in mass rearing and offer a theoretical basis for improving the field performance of *E. sophia*.

## Simple Summary

The facultative autoparasitoid *Encarsia sophia* (Hymenoptera, Aphelinidae) is an important biological control agent against whiteflies including *Bemisia tabaci* (Hemiptera: Aleyrodidae), *Trialeurodes vaporariorum* (Hemiptera: Aleyrodidae) and *Aleurocybotus indicus* (Hemiptera: Aleyrodidae). This study demonstrates that secondary host selection significantly influences offspring fitness and population growth parameters. When male *E. sophia* were reared on the heterospecific host *E. formosa* instead of conspecifics, the resulting females exhibited shorter developmental times, extended adult longevity, and increased fecundity. These females also displayed improved host-feeding capacity and higher killing rates of *B. tabaci* nymphs. Population projection models revealed a dramatic increase in population growth and pest-suppression potential in wasps derived from the heterospecific rearing system. These findings provide valuable insights for optimizing mass-rearing protocols and improving the efficacy of *E. sophia* in biological control applications.

## 1. Introduction

Hyperparasitoids are insects in the fourth trophic level that develop obligately on primary parasitoids [1,2,3]. While most hyperparasitoids are hymenopterans, with representatives in at least 17 families, a minority belong to the Diptera and Coleoptera families [4,5,6]. Unlike primary parasitoids that complete their development on herbivorous hosts [7,8,9], hyperparasitoids must parasitize primary parasitoid larvae or pupae of primary parasitoids to ensure successful development [4,6,10]. A notable subgroup is the autoparasitoids, which exhibit a sexually dimorphic developmental strategy in which females develop as primary parasitoids on herbivorous hosts via arrhenotokous parthenogenesis, while males develop as hyperparasitoids on conspecific or heterospecific parasitoids [11,12,13]. This reproductive strategy necessitates the elimination of primary parasitoid hosts for male production [14,15,16]. Despite this intraguild predation, field studies have shown that the combined release of autoparasitoids and primary parasitoids can synergistically improve pest suppression [17,18,19].

*E. sophia* (Hymenoptera: Aphelinidae), a facultative autoparasitoid, is an effective biological control agent against several whitefly pests, including *T. vaporariorum*, *B. tabaci*, and *A. indicus* [20,21,22]. *E. sophia* suppresses whitefly populations through both parasitism and host-feeding, with its host-feeding capacity notably surpassing that of *E. formosa* by over threefold, establishing it as dominant parasitoids for whitefly management [9,23,24]. Mated females lay fertilized eggs in whitefly nymphs (primary hosts) to produce female offspring, while both mated and unmated females deposit unfertilized eggs in larvae or pupae of conspecific or heterospecific parasitoids (secondary hosts) to produce male offspring [16,25]. The choice of primary and secondary hosts during mass rearing critically influences the body size and biological control potential of *E. sophia* [15,26,27]. Previous studies identified *T. vaporariorum* as the optimal primary host and mature larvae of *E. formosa* as the preferred secondary host, with resultant females exhibiting significantly larger body sizes and enhanced biocontrol efficacy compared to those reared on *B. tabaci* as a primary host or conspecific females as a secondary host [15,16,26].

Facultative autoparasitism serves as both a self-regulatory mechanism for population density and a competitive strategy against heterospecific parasitoids [24,28]. By hyperparasitizing heterospecific larvae or pupae to produce males, facultative autoparasitoids can suppress competitor populations and gain ecological dominance [15]. Empirical evidence reveals a general preference for heterospecific secondary hosts among facultative autoparasitoids [10,29,30]. For instance, *E. tricolor* preferentially selects *E. formosa* as secondary hosts, while *E. pergandiella* demonstrates higher oviposition rates on heterospecific hosts than on conspecifics [31,32]. Although *E. sophia* also exhibits a strong preference for *E. formosa* larvae as secondary hosts [15], the specific impacts of using a heterospecific hosts on the fitness of male offspring and the subsequent reproductive performance of the females they sire remain unclear.

Accurately assessing these impacts requires robust demographic tools. Traditional female-based life tables are inadequate for this purpose, as they exclude male contributions and can misrepresent population dynamics [33,34,35]. Chi and colleagues [36,37] quantified the methodological bias resulting from exclusion of males in population parameter estimation and subsequently developed the age-stage, two-sex life table theory. This framework, supported by specialized software (TWOSEX-MSChart, CONSUME-MSChart, and TIMING-MSChart) [38,39,40], is now widely adopted in entomology [34,41,42]. This framework enables comprehensive evaluation of natural enemy biocontrol potential and population trajectory forecasting via TIMING-MSChart [43,44,45,46]. Beyond advancing insect population ecology, these tools provide foundational support for mass-rearing optimization and successful implementation of biocontrol strategies [47]. By quantifying survival, stage differentiation, fecundity, and parasitism efficiency, two-sex life tables provide a foundational tool for optimizing mass-rearing and enhancing the precision of biocontrol programs.

To elucidate the impact of secondary host selection on offspring fitness in the facultative autoparasitoid *E. sophia*, we established two populations with males reared on heterospecific (*E. formosa*) or conspecific secondary hosts. We measured developmental duration and post-mating pest-suppression capacity in both sexes. Furthermore, we analyzed population parameters using TWOSEX-MSChart, host consumption rates using CONSUME-MSChart, and temporal dynamics using TIMING-MSChart to predict demographic trajectories [36,48,49]. This systematic approach provides actionable insights for optimizing *E. sophia* mass-rearing and field deployment strategies.

## 2. Materials and Methods

### 2.1. Host Plants

Tomato seeds, (cv. Ruiqi I, Xi’an Qunxing Seed Co., Ltd., Xi’an, Shaanxi, China, 2023) *Lycopersicon esculentum* Mill., were sown in seedling trays containing nutrient soil (Baishan Shenzhizhibei Agricultural Technology Co., Ltd., Baishan, Jilin, China, 2023). Seedlings were transplanted into pots (height 15 cm, diameter 16 cm, volume 1.5 L) upon developing the 4th true leaf and cultivated until reaching 7–8 true leaves for experimentation.

### 2.2. Insects

*B. tabaci* (MED cryptic species): collected in 2022 from tomato leaves in Changchun Lianhua Mountain greenhouse (43°48′53.39″ N, 125°26′10.49″ E). Mitochondrial cytochrome oxidase I (mtCOI) gene sequences were confirmed via Sanger sequencing of PCR-amplified products using primers and protocols from Xu et al. [50]. Continuously reared on tomato plants for over 10 generations, *E. formosa* were collected in 2022 from tomato leaves in the Jilin Agricultural University greenhouse (43°80′86.04″ N, 125°41′23.80″ E; host: *T. vaporariorum*). Rearing was maintained for over five generations using tomato and *B. tabaci*. *E. sophia* were collected in 2022 from pepper leaves in the Guangdong Academy of Agricultural Sciences experimental field (23°15′11.20″ N, 113°35′90.90″ E; host: *B. tabaci*), which were continuously reared for over 10 generations using *B. tabaci* as the primary host (secondary host: *E. formosa* or *E. sophia*). For this study, the rearing of male *E. sophia* involved host plants (tomato), pests (*B. tabaci*), primary parasitoids (*E. sophia* and *E. formosa*), and male hyperparasitoids (reared using 3rd-instar larvae of *E. formosa* and *E. sophia* as secondary hosts). The abbreviation ES(ef) was designated for the *E. sophia* population with males reared on *E. formosa* as secondary host, and ES(es) for the population with males reared on as secondary host, thus defining the abbreviations by the secondary host of the males. All insects and plants were maintained in artificial climate chambers (Model YCS-20, from Nanjing Shiheng Bath Instrument Equipment Co., Ltd. in Nanjing, Jiangsu, China) under 16L:8D photoperiod, temperature (26 ± 1) °C, and RH (60 ± 5)%.

### 2.3. Primary and Secondary Hosts

Primary hosts (*B. tabaci* nymphs): micro-cages 4.0 cm (diam.) × 3.5 cm (high) were clipped onto tomato leaf abaxial surfaces [51]. Twenty-five pairs of *B. tabaci* adults were aspirated into micro-cages and removed after 24 h. When nymphs reached the third instars, excess nymphs were removed under a stereomicroscope using insect pins, retaining 60 third-instars nymphs per leaf. Infested leaves were prepared for hydroponic cultivation following Zhao et al. [16]. The *B. tabaci* eggs were allowed to develop to the second and third instar and were then used in the experiments as primary hosts.

Heterospecific secondary hosts (*E. formosa* as secondary host) to reproduce *E. sophia* females and males: tomato leaves with 300 third-instar *B. tabaci* nymphs were hydroponically cultured in insect-rearing containers. Fifteen *E. formosa* adults were introduced and removed after 24 h oviposition. After 5 days of development, nine newly emerged (<6 h) *E. sophia* virgin females were added and removed after 24 h. To enhance readability, we abbreviated *E. sophia* males reared on *E. formosa* as secondary hosts as ESM(ef), and similarly, *E. sophia* females derived from these males as ESF(ef). Males were paired with newly emerged *E. sophia* females (female:male ratio 3:1) in containers with 300 third-instar *B. tabaci* nymphs to produce F1 females. This process was repeated for over 5 generations, ultimately establishing an *E. sophia* population that utilizes *E. formosa* as a secondary host for life table study conspecific secondary hosts (*E. sophia* as secondary host) to reproduce *E. sophia* females and males: tomato leaves with 300 third-instar *B. tabaci* nymphs were hydroponically cultured. Fifteen mated *E. sophia* adults were introduced and removed after 24 h. After 5 days, nine newly emerged (<6 h) *E. sophia* virgin females were added and removed after 24 h. To enhance readability, *E. sophia* males reared on conspecific *E. sophia* as secondary hosts were abbreviated as ESM(es), while *E. sophia* females derived from these males were abbreviated as ESF(es). Males were paired with newly emerged *E. sophia* females (3:1 ratio) to produce F1 females. This process was repeated for over 5 generations, ultimately establishing an *E. sophia* population that utilizes *E. sophia* as a secondary host for life table study. All insects and plants were maintained in artificial climate chambers (Nanjing Hengyu Instrument Equipment Co., Ltd., Model YCS-20) under 16L:8D photoperiod, temperature (26 ± 1) °C, and RH (60 ± 5)%.

### 2.4. Life Table Study: Developmental Duration of Females and Males

Select tomato leaves with approximately 200 third-instar nymphs of *B. tabaci* for hydroponic culture and place them into a hydroponic cup (4.0 cm diameter × 5.6 cm height). Then, select 30 pairs of newly emerged (<6 h) *E sophia* females and males from the crosses ESF(ef) × ESM(ef) and ESF(es) × ESM(es), respectively, and introduce them into different hydroponic cups. After 6 h of oviposition, remove the adult whiteflies. Observe and record the number of parasitized *B. tabaci* nymphs in the two populations under a dissecting microscope, take photos with a computer for marking, and observe once every 24 h. Record the egg–larval period, pupation rate, pupal period, and eclosion rate of each female *E. sophia* wasp (The number of observed eclosed samples should be ≥20).

The tomato leaves with approximately 200 third-instar nymphs of *B. tabaci* are for hydroponic culture, so place them into a hydroponic cup. Then, introduce 20 pairs of *E. sophia* (ESFes × ESMes) adults that have emerged for 2–3 days or 20 *E. formosa* adults that have emerged for 2–3 days into hydroponic cup, respectively. After 6 h of oviposition, remove the adult wasps. Five days after development, introduce 20 newly emerged (<6 h) virgin female *E. sophia* (ESFes and ESFef) into each cup, and remove them after 6 h of oviposition. After approximately 5 days of development, observe and record the number of parasitized *E. sophia* larvae in the two populations under a dissecting microscope, take photos with a computer for marking, and observe once every 24 h. Record the egg–larval period, pupation rate, pupal period, and eclosion rate of each male *E. sophia* wasp (the number of observed eclosed samples should be ≥20).

### 2.5. Reproduction and Pest Control Capacity Assays

Adults of both sexes that had newly emerged in the life table study were collected. Twenty pairs of newly emerged (<6 h) ESF(ef) × ESM(ef) and ESF (es) × ESM (es) were separately introduced into containers (20 groups per population) with tomato leaves bearing 60 third-instar *B. tabaci* nymphs. Leaves were replaced every 24 h. Removed leaves were stored in marked tubes with water-moistened cotton-wrapped petioles. After 5 days, parasitized and consumed nymphs were microscopically quantified until female wasp death (no male replacement if mortality occurred). All experiments were conducted in an artificial climate chamber (Nanjing Hengyu Instrument Equipment Co., Ltd., Model: YCS-20) under control 16L:8D photoperiod, temperature (26 ± 1) °C, and RH (60 ± 5)%.

### 2.6. Data Analysis

The stage-specific survival rate (*s_xj_*) at a specific age refers to the probability that newly produced offspring survive to the *x*-day-old and *j*-stage, calculated using the method proposed by Chi et al. [34,36,37]. The simplified formula for calculating *s_xj_* is as follows:

(1) $$s_{xj}=\frac{n_{xj}}{n_{01}}\;$$

where *n*_01_ is the number of newly emerged *E. sophia* used at the start of the study, and *n_xj_* is the number of individuals among *n*_01_ that survive to the *x*-day-old and *j*-stage. The formulas for the age-specific survival rate (*l_x_*) and age-specific fecundity (*m_x_*) are as follows:

(2) $$l_{x}=\sum_{j=1}^{\beta }s_{xj}\;$$

(3) $$m_{x}=\frac{\sum_{j=1}^{\beta }s_{xj}f_{xj}}{\sum_{j=1}^{\beta }s_{xj}}$$

According to Mou et al. [52], the net reproductive rate (*R*_0_) refers to the average number of offspring produced by a parasitoid during its lifetime, calculated as follows:

(4) $$R_{0}=\sum_{x=0}^{\infty }l_{x}m_{x}$$

The intrinsic rate of increase (*r*) is calculated using the iterative bisection method based on the Euler–Lotka equation [53], with age counted from day 0. The formulas for the finite rate of increase (*λ*) and mean generation time (*T*) are as follows:

(5) $$\sum_{x=0}^{\infty }e^{-r\left(x+1\right)}l_{x}m_{x}=1$$

(6) $$\lambda =e^{r}$$

(7) $$T=\frac{\ln R_{0}}{r}$$

The age-stage life expectancy (*e_xj_*) is calculated as follows:

(8) $$e_{xj}=\sum_{i=x}^{\infty }\sum_{y=j}^{\beta }{s^{\prime }}_{iy}$$

where *s_iy_*′ is the probability that *n_xj_* individuals survive to the *i*-day-old and *y*-stage, assuming *s_xj_* =1. The age-stage reproductive value (*v_xj_*) is calculated using the method of Tuan et al. [54]:

(9) $$v_{xj}=\frac{e^{r\left(x+1\right)}}{s_{xj}}\sum_{i=x}^{\infty }e^{-r\left(i+1\right)}\sum_{y=j}^{\beta }{s^{\prime }}_{iy}f_{iy}$$

The age-specific host consumption rate (*k_x_*) is calculated as follows:

(10) $$k_{x}=\frac{\sum_{j=1}^{\beta }s_{xj}c_{xj}}{\sum_{j=1}^{\beta }s_{xj}}$$

where *c_xj_* is the consumption rate of parasitoids at the *x*-day-old and *j*-stage. According to the method of Yu et al. [55], considering the age-specific survival rate, the age-specific net host consumption rate (*q_x_*) is calculated as follows:

(11) $$q_{x}=l_{x}k_{x}=\sum_{j=1}^{\beta }s_{xj}c_{xj}$$

The net host consumption rate (*C*_0_), representing the total number of hosts consumed by adult parasitoids during their lifetime, is calculated as follows:

(12) $$C_{0}=\sum_{x=0}^{\infty }l_{x}k_{x}=\sum_{x=0}^{\infty }\sum_{j=1}^{\beta }s_{xj}c_{xj}$$

The finite host consumption rate (*ω*) is calculated as follows:

(13) $$\omega =\lambda \psi =\lambda \sum_{x=0}^{\infty }\sum_{j=1}^{\beta }a_{xj}c_{xj}$$

where *a_xj_* is the stable age-stage distribution, and *ψ* is the stable host consumption rate, calculated as follows:

(14) $$\psi =\sum_{x=0}^{\infty }\sum_{j=1}^{\beta }a_{xj}c_{xj}$$

The stage-specific kill rate (*d_xj_*) is the sum of the total number of whiteflies killed by parasitism (*f_xj_*) and consumption (*c_xj_*). Thus, *d_xj_* = *f_xj_* + *c_xj_*. The formulas for the age-specific kill rate (*u_x_*) and age-specific net predation kill rate (*w_x_*) are as follows:

(15) $$u_{x}=\frac{\sum_{j=1}^{\beta }s_{xj}\left(f_{xj}+c_{xj}\right)}{\sum_{j=1}^{\beta }s_{xj}}=\frac{\sum_{j=1}^{\beta }s_{xj}d_{xj}}{\sum_{j=1}^{\beta }s_{xj}}$$

(16) $$w_{x}=l_{x}u_{x}$$

The net kill rate (*Z*_0_) is calculated as follows:

(17) $$Z_{0}=\sum_{x=0}^{\infty }l_{x}u_{x}=\sum_{x=0}^{\infty }\sum_{j=1}^{\beta }s_{xj}\left(f_{xj}+c_{xj}\right)$$

The formulas for the stable kill rate (*θ*) and finite kill rate (*υ*) are as follows:

(18) $$\theta =\sum_{x=0}^{\infty }\sum_{j=1}^{\beta }a_{xj}d_{xj}$$

(19) $$\upsilon =\lambda \theta =\lambda \sum_{x=0}^{\infty }\sum_{j=1}^{\beta }a_{xj}d_{xj}$$

The conversion rate (*Q_p_*), defined as the total number of hosts killed (including consumption and parasitism) to produce a single parasitoid offspring, is calculated as follows:

(20) $$Q_{p}=\frac{Z_{0}}{R_{0}}=\frac{R_{0}+C_{0}}{R_{0}}$$

The TIMING-MSChart computer program [40] was used to predict the reproductive and pest control potential of *E. Sophia*, where *n_xj_* (*t*) is the number of *E. sophia* individuals at *x*-day-old and *j*-stage at time *t*. The formula for pest control potential at time *t* is as follows:

(21) $$K\left(t\right)=\sum_{j=1}^{\beta }\sum_{x=0}^{\infty }d_{xj}n_{xj}\left(t\right)$$

To estimate the variance and standard error of all parameters, we employed the bootstrap technique embedded in TWOSEX-MSChart and COMSUME-MSChart, with *B* = 100,000 resampling iterations. Paired bootstrap tests were used to evaluate the significance of differences between treatments based on the 95% percentile confidence intervals and t-intervals of 100,000 differences [48,56]. The population growth of *E. sophia* was predicted using the computer program TIMING-MSChart, based on the theory proposed by Chi [40].

## 3. Results

### 3.1. Life Table of E. sophia Reproduced by Different Secondary Hosts

The developmental times of eggs to larvae, pupae, and pre-adult duration of both female and male *E. sophia* were significantly shorter in the ES(ef) population compared to the ES(es) population (Table 1). Meanwhile, the adult longevity of both females and males was significantly longer in the ES(ef) population. However, due to the shorter pre-adult developmental time in the ES(ef) population, there was no significant difference in the total lifespan between the two populations for both sexes. There were no significant differences in the APOP (adult preoviposition period) and TPOP (total preoviposition period) between females of the two populations. Nevertheless, the oviposition period of females in the ES(ef) population was significantly longer than that of the ES(es) population, and they exhibited a higher mean fecundity (103.55 offspring/female) (Table 1).

**Figure 1:**
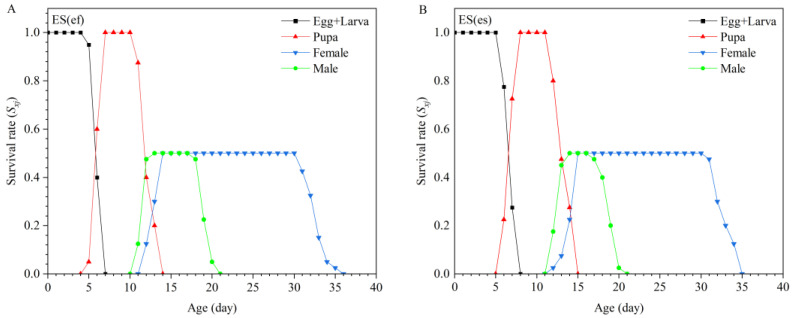
Age-stage survival rate (*S_xj_*) of ES(ef) population (**A**) and ES(es) population (**B**).

When considering the age-specific survival rates (*S_xj_*) of *E. sophia* from both populations, in the ES(ef) population, males began to emerge at 10 days old, peaking at 11–12 days, while females started emerging at 11 days old, with most emerging between 12 and 14 days. In contrast, in the ES(es) population, males began emerging at 11 days old, peaking at 12–13 days, and females started emerging at 11 days old, with the highest emergence occurring at 14–15 days (Figure 1).

**Figure 2:**
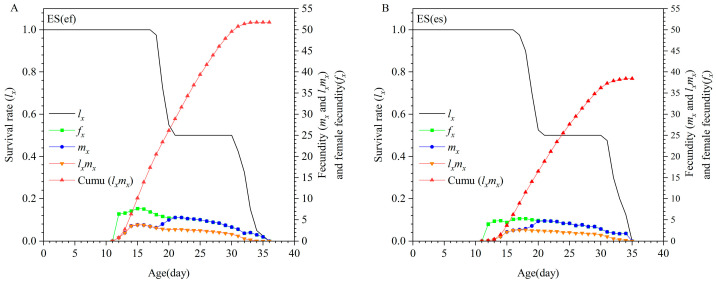
Age-specific survival rate (*l_x_*), female fecundity (*f_x_*), age-specific fecundity (*m_x_*), and age-specific net maternity (*l_x_m_x_*) of ES(ef) population (**A**) and ES(es) population (**B**).

Females in the ES(ef) population reached their peak oviposition rate at 15 days old (7.65 offspring/female), which was significantly higher than the peak oviposition rate of females in the ES(es) population at 18 days old (5.3 offspring/female). Furthermore, the age-specific fertility (*m_x_*), net reproductive rate at a given age (*l_x_m_x_*), and cumulative net reproductive rate Cumu(*l_x_m_x_*)] were all significantly higher in the ES(ef) population compared to the ES(es) population (Figure 2).

**Figure 3:**
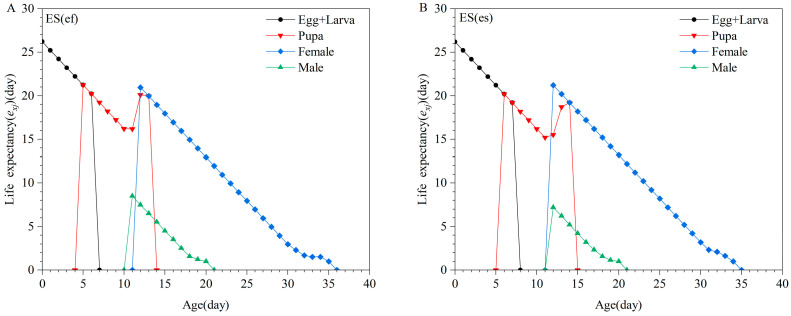
Life expectancy (*e_xj_*) of ES(ef) population (**A**) and ES(es) population (**B**).

**Figure 4:**
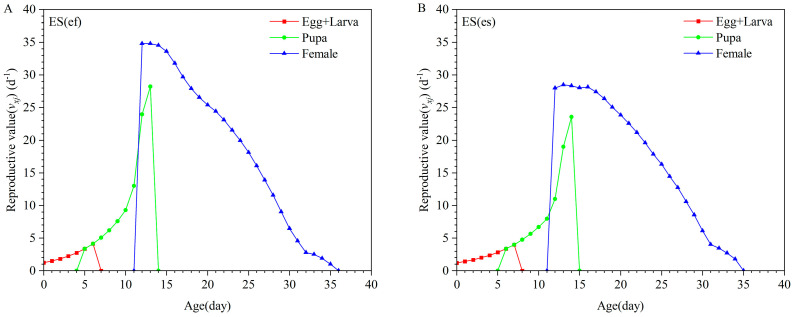
Reproductive value (*v_xj_*) of ES(ef) population (**A**) and ES(es) population (**B**).

There were no significant differences in the life expectancy (*e_xj_*) of larvae, pupae, and females between the two populations. However, the longevity of males in the ES(ef) population was significantly longer than that of males in the ES(es) population (Figure 3). The reproductive value (*v_xj_*) of females in the ES(ef) population peaked at 12 days old at 34.82 offspring/day, whereas the peak for females in the ES(es) population was at 13 days old at 28.52 offspring/day (Figure 4).

The net reproductive rate (*R*_0_) of the ES(ef) population was 51.78 offspring, which was not significantly different from the *R*_0_ of the ES(es) population at 38.45 offspring. However, the intrinsic rate of increase (*r*) and finite rate of increase (*λ*) were significantly higher in the ES(ef) population compared to the ES(es) population, and the mean generation time (*T*) was significantly shorter (Table 2).

### 3.2. Host-Feeding Rate and Pest-Killing Rate

**Figure 5:**
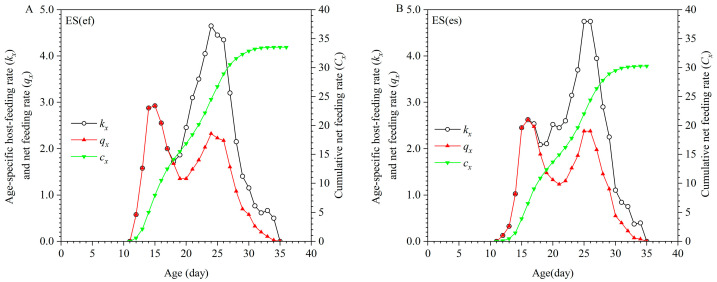
Age-specific host-feeding rate (*k_x_*), age-specific net host-feeding rate (*q_x_*), and cumulative host-feeding rate (*C_x_*) of ES(ef) population (**A**) and ES(es) population (**B**).

During the larval stage, *E. sophia* develops inside whitefly nymphs and cannot feed on them, resulting in zero values for age-specific host-feeding rate (*k_x_*), age-specific net host-feeding rate (*q_x_*), and cumulative host-feeding rate (*C_x_*) until emergence. Females in the ES(ef) population reached the peak *k_x_* value at 24 days old at 4.65 nymphs/female, while females in the ES(es) population peaked at 25 days old at 4.75 nymphs/female. The *k_x_* and *q_x_* values remained consistent until 17 days old in the ES(ef) population, when male mortality began, causing a divergence; likewise, in the ES(es) population, *k_x_* and *q_x_* values remained aligned until 16 days old (Figure 5).

**Figure 6:**
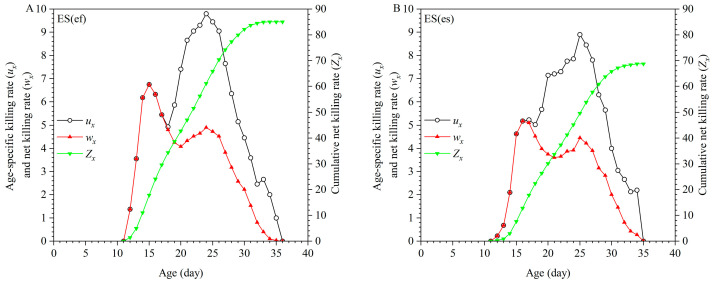
Age-specific whitefly-killing rate (*u_x_*), age-specific net killing rate (*w_x_*), and cumulative killing rate (*Z_x_*) of ES(ef) population (**A**) and ES(es) population (**B**).

The age-specific whitefly-killing rate (*u_x_*), of *E. sophia* in the ES(ef) population reached a maximum of 9.8 nymphs/individual at 24 days old, which was significantly greater than the maximum *u_x_* of 8.9 nymphs/individual in the ES(es) population at 25 days old. Due to the shorter longevity of males compared to females, the age-specific net killing rate (*w_x_*) in both populations diverged from *u_x_* when males began to die, at 18 days old in ES(ef) and 17 days old in ES(es), with peak *w_x_* values occurring at 15 days old (6.75 nymphs/individual) and 16 days old (5.18 nymphs/individual), respectively (Figure 6).

The net host-feeding rate (*C*_0_) of *E. sophia* showed no significant differences between the two populations. However, the per-female host-feeding rate of the ES(ef) population was 67.05 individuals, which was significantly higher than that of the ES(es) population. There were no significant differences in the stable host-feeding rate (*ψ*) or finite host-feeding rate (*ω*) of *E. sophia* between the ES(ef) and ES(es) populations. The female host-killing rates were 170.60 nymphs/female and 137.45 nymphs/female for the ES(ef) and ES(es) populations, respectively, with a significant difference between them. The net killing rate (*Z*_0_), stable killing rate (*θ*), and finite killing rate (*υ*) were all higher in the ES(ef) population compared to the ES(es) population. Conversely, the transformation rate (*Q_p_*) of *E. sophia* in the ES(ef) population was 1.64, which was significantly lower than the *Q_p_* of 1.79 in the ES(es) population(Table 3).

### 3.3. Population Projection

**Figure 7:**
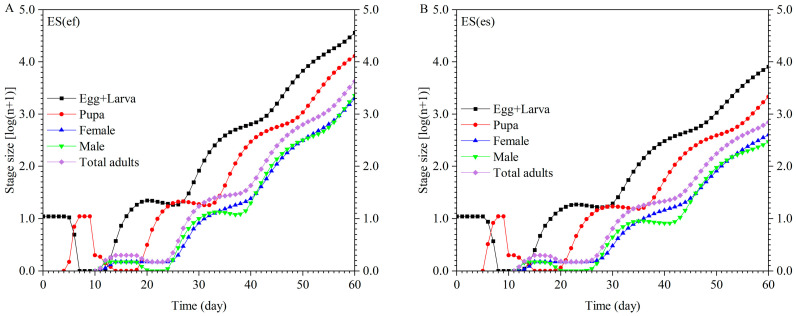
Population projection of ES(ef) population (**A**) and ES(es) population (**B**).

Using the survival, development, and fecundity data obtained from these experiments results, the population growth of both populations was predicted using TIMING-MSChart software (Figure 7). Starting with 10 *E. sophia* eggs for both populations, after 60 days of reproduction, the ES(ef) population consisted of 36,374.55 eggs + larvae, 12,927.13 pupae, 1988.79 female adults, and 2284.04 male adults. In contrast, the ES(es) population had 8096.90 eggs + larvae, 2152.13 pupae, 399.75 female adults, and 294.49 male adults. The growth rate of *E. sophia* in the ES(ef) population was significantly faster than that of the ES(es) population, with significantly higher numbers of individuals at all stages on day 60.

**Figure 8:**
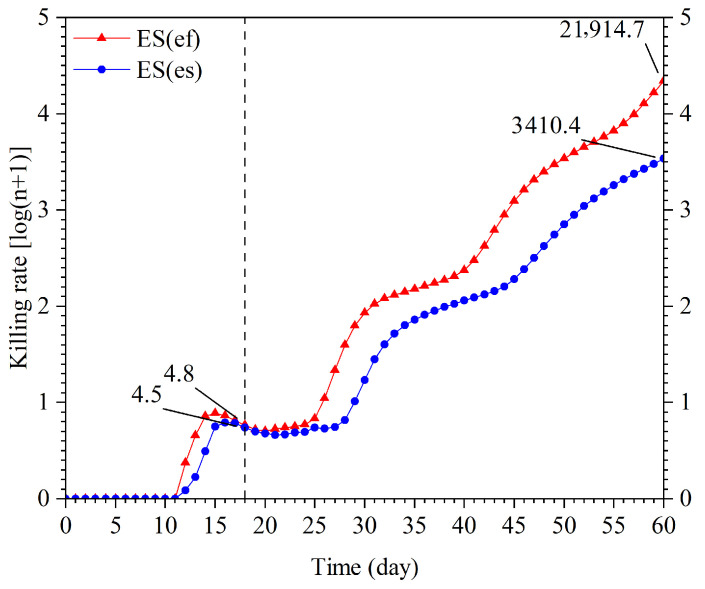
Projection of total killing rate of ES(ef) population and ES(es) population.

Combining these prediction data with the host-killing rates of *E. sophia* from both populations, the number of host nymphs killed by each population was predicted using TIMING-MSChart software (Figure 8). In the first 25 days, the number of nymphs killed by both populations was similar, with the closest match occurring on day 18. As the population sizes continued to grow, the rate of increase in the number of nymphs killed by the ES(ef) population was significantly higher than that of the ES(es) population. By day 60, the ES(ef) population had killed approximately 21,914.7 nymphs, which was approximately six times the number killed by the ES(es) population (3410.5 nymphs).

## 4. Discussion

The age-stage, two-sex life table is a robust demographic tool that effectively integrates the reproductive contributions of male individuals while accurately characterizing the developmental differentiation between sexes [14,57]. This methodology has been widely applied in entomological ecological research to derive precise population parameters for various insect species [37,58]. Insect population parameters serve as fundamental indicators for optimizing mass-rearing protocols of beneficial insects and developing evidence-based biological control strategies [34]. Recent studies have increasingly adopted the age-stage, two-sex life table approach to analyze population parameters of biological control agents, enabling the development of more precise and effective pest management strategies [43,59,60].

Previous studies have established that parasitoid wasp developmental duration is modulated by multiple host factors, including species identity, instar stage, and body size of both primary and secondary hosts [61,62,63]. A particularly interesting case is *E. sophia*, which demonstrates a host preference for larvae or pupae of heterospecific parasitoid wasps, with mature *E. formosa* larvae serving as the predominant secondary host [15]. Notably, *E. sophia* males reared on *E. formosa* exhibited significantly shorter developmental durations compared to those developing from conspecific females [9,15]. Parallel studies have revealed similar sex-specific variations in other biological control agents. Cao et al. [64] systematically investigated the relationship between developmental time, longevity, and pest control efficacy in *Arma chinensis*, while Park et al. [65] demonstrated that the predatory mite *Gaeolaelaps aculeifer* possesses a shorter mean generation time than *Stratiolaelaps scimitus*, suggesting superior biocontrol potential. The significant difference in male development times between the two populations ES(ef) and ES(es) of *E. sophia* in this study aligns with the findings of Zang et al. [15]. This study found significant developmental divergence between the ES(ef) and ES(es) populations of *E. sophia*. Specifically, female *E. sophia* from the ES(ef) population developed significantly faster than their ES(es) counterparts. Adult longevity in the ES(ef) population was 19.80 days for females and 7.70 days for males, significantly longer than the 18.85 days and 6.45 days recorded for females and males in the ES(es) population, respectively. However, no significant differences in total longevity were observed between the two populations for either sex. Collectively, the ES(ef) population exhibited dual advantages, accelerated development and extended adult longevity in both sexes, thereby shortening generation cycles. This combination likely enhances population recruitment rates and pest-suppression capacity, enabling rapid pest population regulation [9,14].

Parasitic wasps primarily suppress pests by parasitizing, consuming, and killing host pests during the larval stage [4,9]. Zang et al. [44] utilized the TWOSEX-MSChart software to analyze fecundity, oviposition period, and APOP data to compare the biological control potential of three species of *Anastatus*. Zang et al. [15] found that when female *E. sophia* mated with males reproduced from *E. formosa* as a secondary host, the females had a longer oviposition period and higher fecundity compared to those mated with males bred from conspecific females. This trend is consistent with the significant differences in fecundity and oviposition period observed between the two populations in this study. Xu et al. [14] reported an APOP of 0.13 days for *E. sophia*, whereas no APOP was observed for females from both populations in this study, potentially due to differences in host plants leading to a higher number of mature eggs in these females. The TPOP for females in the ES(ef) population was significantly shorter than that of females in the ES(es) population. Additionally, the oviposition period of 19.60 days for ES(ef) females was significantly longer than the 18.50 days for ES(es) females, and also significantly longer than the 18.50 days reported by Xu et al. [14] for *E. sophia* and the 16.60 days reported by Zhao et al. [51] for *E. formosa*. Although there was no significant difference in net reproductive rate, the ES(ef) population had significantly higher fecundity, intrinsic rate of increase, and finite rate of increase compared to the ES(es) population. This could be attributed to the semen of males reproduced from *E. formosa* containing more nutrients, providing nutritional supplements to the mating females, which may increase the number of mature eggs and enhance fecundity [66]. Furthermore, *E. formosa* harbors the symbiotic bacterium *Wolbachia*, which provides nutritional support [67]. Whether *E. sophia* reproduced using *E. formosa* as a secondary host contains *Wolbachia*, thereby providing additional nutrients, requires further investigation.

Apart from parasitism, whitefly parasitic wasps can directly feed on and kill whitefly nymphs, the host-feeding ability is also one of the critical data for evaluating the pest control potential of biological control insects [9,68,69]. Numerous studies have used CONSUME-MSChart software to analyze host-feeding quantities for assessing the efficacy of natural enemies [44,51,70]. *E. sophia* exhibits exceptional host-feeding capacity, with its feeding-induced mortality of whitefly nymphs comparable to parasitism-induced mortality, and three times higher than that of *E. formosa* and *E. hayati* [14,24]. Thus, feeding quantity constitutes a vital parameter for evaluating the biocontrol potential of *E. sophia*. In this study, the net host-feeding rate of the ES(ef) population showed no significant difference from the ES(es) population. However, the per-female host-feeding rate of ES(ef) was significantly higher than that of ES(es). This discrepancy arises because *E. sophia* males cannot independently feed on whitefly nymphs, being limited to consuming body fluids exuded from nymphs previously probed by female ovipositors [71].

Comparing the total number of whitefly pests killed by parasitoids can more objectively and accurately evaluate their potential for biological control [14,16,51]. Zhao et al. [51] used CONSUME-MSChart software to determine that the net kill number of *E. formosa* reared on yacon for whitefly nymphs was 239.73 nymphs/female, significantly higher than the 200.20 nymphs/female for *E. formosa* reared on tomato. But there was no significant difference in net killing rates between the two populations. On the contrary, the female-killing rate of the ES(ef) population (170.60 nymphs/female) was significantly higher than that of the ES(es) population (137.45 nymphs/female). The discrepancy in significance between net and female-killing rate is due to the fact that only female *E. sophia* can kill whitefly nymphs through parasitism and host-feeding, while males do not directly cause nymph mortality [71]. Collectively, these findings indicate that the ES(ef) population demonstrates superior control capabilities against whitefly pests, outperforming the ES(es) population in both parasitism and host-feeding efficiency.

The stable killing rate and the finite killing can be employed to compare the biological control potential of two populations against whitefly nymphs [14,51]. This study found that there was no significant difference in the stable killing rate and the finite killing between the ES(ef) population and the ES(es) population. However, as the stable killing rate and the finite killing approach infinity over time, the population will form a stable age-stage distribution, thus limiting their practical applications [51]. Zhao et al. [51] used the TIMING-MSChart software to simulate the population growth of *E. formosa* reared on different host plants and the number of whitefly nymphs killed by these populations. Similarly, Cao et al. [64] utilized software to simulate the population growth of *Arma chinensis*. In this study, we employed TIMING-MSChart [40] to predict population growth trends and whitefly nymph elimination capacities of two *E. sophia* populations (Figure 7 and Figure 8). The results clearly demonstrate that the ES(ef) population exhibits faster population growth and greater pest control potential against whitefly nymphs compared to the ES(es) population. Previous studies on facultative autoparasitoid secondary hosts mainly focused on host selection aspects, with only Zang et al. [15] investigating secondary host effects on male production and subsequent mating females. Using age-stage, two-sex life tables, this study systematically revealed how secondary hosts influence male developmental duration and longevity in *E. sophia*, as well as the developmental duration, longevity, and pest control capacity of mated females.

## 5. Conclusions

This study provides clear evidence that secondary host selection significantly shapes the life history parameters and biocontrol potential of *E. sophia*. When males were reared on the heterospecific host *E. formosa*, the resulting individuals exhibited shortened developmental times, increased adult longevity, higher fecundity, and improved host-feeding performance compared to those developed from conspecific hosts. Population projections based on age-stage two-sex life table analysis demonstrated faster population growth and superior whitefly suppression capacity in the heterospecific-reared population. These results provide theoretical guidance for efficient mass rearing of *E. sophia* and establish a foundation for its practical application in controlling whitefly pests.

## Figures and Tables

**Table 1:** Developmental times (means ± SE), adult longevity, total longevity, total preoviposition period (TPOP), oviposition days, and fecundity of the ES(ef) population and the ES(es) population.

Parameter	Population				*p*-Value
	*n*	ES(ef)	*n*	ES(es)	
Female egg–larva duration (days)	20	6.70 ± 0.10 b	20	7.45 ± 0.15a	<0.0001
Male egg–larva duration (days)	20	6.00 ± 0.10 b	20	6.65 ± 0.11a	<0.0001
Female pupa duration (days)	20	6.45 ± 0.11 b	20	6.90 ± 0.07a	0.0008
Male pupa duration (days)	20	5.80 ± 0.09 b	20	6.10 ± 0.07a	0.0101
Female total pre-adult duration (days)	20	13.15 ± 0.18 b	20	14.35 ± 0.19a	<0.0001
Male total pre-adult duration (days)	20	11.80 ± 0.12 b	20	12.75 ± 0.14a	<0.0001
Female adult longevity (days)	20	19.80 ± 0.22 a	20	18.85 ± 0.31b	0.0136
Male adult longevity (days)	20	7.70 ± 0.13 a	20	6.45 ± 0.17b	<0.0001
Female total longevity (days)	20	32.95 ± 0.29 a	20	33.20 ± 0.29a	0.5450
Male total longevity (days)	20	19.50 ± 0.18 a	20	19.20 ± 0.21a	0.2671
APOP (days) ^1^	20	0.00	20	0.00	1.0000
TPOP (days) ^2^	20	13.15 ± 0.19 a	20	14.35 ± 0.19a	<0.0001
Oviposition days (*O_d_*)	20	19.60 ± 0.24 a	20	18.50 ± 0.35b	0.0092
Fecundity (*F*) (eggs/female)	20	103.55 ± 1.94 a	20	76.90 ± 1.95b	<0.0001

Note: Different lowercase letters within the same row indicate significant differences in means (paired bootstrap test, *B* = 100 000, *p* < 0.05). ^1^ Adult preoviposition period. ^2^ Total preoviposition period.

**Table 2:** Population parameters (means ± SE) of different population.

Parameter	Population		*p*-Value
	ES(ef)	ES(es)	
Net reproduction rate (*R*_0_) (offspring)	51.78 ± 8.29 a	38.45 ± 6.18 a	0.2004
Intrinsic rate of increase (*r*) (day^−1^)	0.20 ± 0.01 a	0.17 ± 0.01 b	0.0261
Finite rate of increase (*λ*) (day^−1^)	1.22 ± 0.01 a	1.19 ± 0.01 b	0.0258
Mean generation time (*T*) (days)	19.48 ± 0.22 b	21.08 ± 0.24 a	<0.0001

Note: Different lowercase letters within the same row indicate significant differences in means (paired bootstrap test, *B* = 100 000, *p* < 0.05).

**Table 3:** Parameters of parasitism, feeding, and killing (mean ± SE) of different population.

Parameter	Population		*p*-Value
	ES(ef)	ES(ef)	
Net host-feeding rate (*C*_0_)	33.53 ± 5.33 a	30.25 ± 4.83 a	0.6537
Female host-feeding rate (whiteflies/parasitoid)	67.05 ± 1.07 a	60.50 ± 1.17 b	0.0002
Stable host-feeding rate (*ψ*)	0.16 ± 0.02 a	0.17 ± 0.02 a	0.8183
Finite host-feeding rate (*ω*)	0.19 ± 0.03 a	0.20 ± 0.03 a	0.9384
Net killing rate (*Z*_0_) (whiteflies/parasitoid)	85.05 ± 13.56 a	68.70 ± 10.96 a	0.3519
Female-killing rate (whiteflies/parasitoid)	170.60 ± 2.79 a	137.45 ± 2.55 b	0.0001
Stable killing rate (*θ*)	0.38 ± 0.05 a	0.36 ± 0.05 a	0.7448
Finite killing rate (*υ*)	0.47 ± 0.07 a	0.43 ± 0.06 a	0.6547
Transformation rate (*Q_p_*)	1.64 ± 0.01 b	1.79 ± 0.02 a	<0.0001

Note: Different lowercase letters within the same row indicate significant differences in means (paired bootstrap test, *B* = 100 000, *p* < 0.05).

## Data Availability

The raw data supporting the conclusions of this article will be made available by the authors on request.
